# Acute changes in sexual function, hormonal profiles, and psychological status following intensity-modulated radiotherapy in male patients with nasopharyngeal carcinoma

**DOI:** 10.3389/fonc.2026.1832794

**Published:** 2026-06-02

**Authors:** Yan Xiong, Weiying Deng, Yanxian Tan, Shaoli He

**Affiliations:** Department of Oncology, The First People's Hospital of Foshan (Foshan Hospital Affiliated to Southern University of Science and Technology), School of Medicine, Southern University of Science and Technology, Foshan, Guangdong, China

**Keywords:** intensity-modulated radiotherapy, nasopharyngeal carcinoma, psychological status, sex hormones, sexual dysfunction

## Abstract

**Purpose:**

To evaluate the acute effects of intensity-modulated radiotherapy (IMRT) on sexual function, sex hormone levels, and psychological status in male patients with nasopharyngeal carcinoma, and to further assess the associations between changes in sexual function and hormonal and psychological parameters.

**Methods:**

Eighty-five male patients with nasopharyngeal carcinoma who received IMRT were retrospectively included. Erectile function was evaluated using the International Index of Erectile Function-5 (IIEF-5), and ejaculatory function was assessed using the Chinese Index of Premature Ejaculation (CIPE). Serum sex hormone levels, including follicle-stimulating hormone (FSH), luteinizing hormone (LH), testosterone (T), estradiol (E2), prolactin (PRL), and progesterone (P), as well as psychological status assessed by the Self-Rating Anxiety Scale (SAS) and Self-Rating Depression Scale (SDS), were extracted from clinical records at baseline and 30 days after radiotherapy. Paired comparisons (paired t-tests or Wilcoxon signed-rank tests) and Pearson correlation analyses were performed.

**Results:**

Within 30 days following IMRT, both IIEF-5 and CIPE scores significantly decreased compared with baseline (both p < 0.001). Post-treatment levels of FSH and PRL were significantly increased, whereas T and P levels were significantly decreased (all p < 0.001). No significant changes were observed in LH or E2 levels. Correlation analyses revealed weak associations between sexual function scores and sex hormone levels (|r| < 0.2). SAS and SDS scores did not change significantly after radiotherapy, and psychological scores showed a weak negative correlation with sexual function scores.

**Conclusion:**

In male patients with nasopharyngeal carcinoma, IMRT is associated with a significant decline in sexual function during the acute phase, accompanied by alterations in sex hormone profiles. Sexual function changes showed weak associations with both hormonal and psychological factors.

## 1 Introduction

Nasopharyngeal carcinoma (NPC) is a malignant tumor arising from the epithelial lining of the nasopharyngeal mucosa. The incidence of NPC shows marked geographic variation worldwide, with substantially higher rates observed in East Asia (particularly China), Southeast Asia, and North Africa compared with Western countries. The male-to-female incidence ratio is approximately 2.5:1 (1).

Intensity-modulated radiation therapy (IMRT) is the main treatment modality for NPC and has significantly improved 5-year survival rates to over 80% (2). Due to the anatomical proximity of the nasopharynx to the hypothalamus and pituitary gland, and the high radiosensitivity of these structures, relatively low radiation doses (approximately 40 Gy) may cause pituitary injury, leading to dysfunction of the hypothalamic–pituitary–gonadal (HPG) axis (3) and the development of erectile dysfunction (ED).

In clinical practice, healthcare professionals often focus primarily on disease management, while sexual function is generally considered only as one aspect of quality of life. In addition, patients may be reluctant to disclose sexual problems, which can lead to underrecognition of these symptoms and a lack of systematic intervention. Previous studies have identified sexual function and intimate relationships as important unmet needs among patients with head and neck cancer (HNC) (4–8). However, most research on post-treatment sexual dysfunction has focused on prostate cancer and gynecological malignancies (9, 10), with limited data available for patients with nasopharyngeal carcinoma (NPC). Consequently, there remains a lack of structured clinical intervention strategies and evidence-based guidance, which may adversely affect patients’ quality of life and psychological well-being (11).

The present study aimed to investigate changes in erectile function, ejaculatory function, sex hormone levels, and psychological status during the acute phase of IMRT in male patients with NPC, and to further analyze the relationships among these variables.

## 2 Methods

### 2.1 Study design and participants

This study was designed as a retrospective observational study. Male patients with pathologically confirmed nasopharyngeal carcinoma (NPC) who received intensity-modulated radiation therapy (IMRT) were included. The prescribed dose to the planning target volume was 2.12 Gy per fraction, with a total dose of 69.96 Gy. All treatment plans were developed according to the standard IMRT protocol for NPC in our institution. Target delineation and dose prescription were performed in accordance with the Guidelines for Target Delineation and Treatment Planning of Nasopharyngeal Carcinoma (NCC/T-RT 009-2021) and ICRU Report 83. All radiotherapy plans were reviewed and approved by experienced radiation oncologists and were verified according to routine clinical procedures before treatment delivery.

Patients were included if they met the following criteria (1): histopathologically confirmed NPC (2); age ≥22 years, married, and living with their spouse (3); treatment-related acute adverse effects had largely resolved at the time of evaluation, with a Karnofsky Performance Status (KPS) score >90. Patients were excluded if they met any of the following criteria (1): presence of severe chronic physical illness or psychiatric disorders (2); local recurrence or distant metastasis after NPC treatment (3); prior history of head and neck radiotherapy or endocrine disorders affecting the hypothalamic–pituitary–gonadal (HPG) axis (4); incomplete clinical data or missing key variables. This study was approved by the Ethics Committee of Foshan First People’s Hospital. Given the retrospective nature of the study, the requirement for informed consent was waived. All data were anonymized to ensure patient privacy. A total of 85 male patients with NPC who received IMRT at our institution between June 2018 and June 2023 were included. The sample size was determined based on the number of eligible cases during the study period. Among them, 83 patients received concurrent chemotherapy, while 2 patients did not.

### 2.2 Treatment protocol

All patients received intensity-modulated radiotherapy (IMRT). Computed tomography (CT) simulation was performed before treatment, and magnetic resonance imaging (MRI) was fused for accurate target delineation. Target volumes included the gross tumor volume (GTV), clinical target volume (CTV), and planning target volume (PTV). Definitions and delineation of target volumes were performed in accordance with the Guidelines for Target Delineation and Treatment Planning of Nasopharyngeal Carcinoma (NCC/T-RT 009-2021) and ICRU Report 83. The prescribed dose was stratified according to tumor stage. The high-risk clinical target volume of the primary tumor (CTV1) was delineated based on the hierarchical target definition principles outlined in the above guidelines, mainly including potential subclinical invasion areas surrounding the primary tumor and the drainage regions of involved lymph nodes. According to tumor stage and general condition, some patients received concurrent chemotherapy, predominantly based on platinum-containing doublet regimens.

### 2.3 Data sources and outcome measures

Clinical and demographic data were obtained from the electronic medical record system and previous clinical assessments. Erectile function (IIEF-5), ejaculatory function (CIPE), anxiety (SAS), depression (SDS), and serum sex hormone levels were collected at two time points: before radiotherapy and 30 days after its completion. Serum hormone measurements included six sex-related indicators, and blood samples were consistently collected between 8:00 and 10:00 a.m. In this study, sexual function was defined based on two dimensions: erectile function and ejaculatory function.

#### 2.3.1 General information and clinical variables

Demographic and clinical variables, including age, educational level, religious belief, and disease stage, were extracted from medical records.

#### 2.3.2 International index of erectile function-5

Erectile function over the previous three months was evaluated using the IIEF-5 questionnaire (12). It consists of five items with a total score ranging from 0 to 25. A total score <22 indicates erectile dysfunction, which is categorized as severe (<7), moderate (8–11), or mild (12–21). The scale has demonstrated good internal consistency, with a Cronbach’s α coefficient >0.8.

#### 2.3.3 Assessment of ejaculatory function using the CIPE questionnaire

Ejaculatory function was evaluated using the CIPE questionnaire (13), which consists of 10 items with a total score ranging from 10 to 50. Scores of 37–50 indicate normal ejaculatory function, 34–36 indicate a borderline state characterized by reduced semen concentration or volume and possible retrograde ejaculation, and 10–33 indicate severe ejaculatory dysfunction. Patients classified as grade II or III were considered to have ejaculatory dysfunction.

#### 2.3.4 Self-rating anxiety scale and self-rating depression scale

Psychological status was assessed using the SAS and SDS, each consisting of 20 items scored on a 4-point Likert scale. Standardized scores were calculated by multiplying raw scores by 1.25. For the SAS, scores of 50–59 indicate mild anxiety, 60–69 indicate moderate anxiety, and ≥70 indicate severe anxiety (14). For the SDS, scores of 53–62 indicate mild depression, 63–72 indicate moderate depression, and ≥73 indicate severe depression (15). The Cronbach’s α coefficient for the scales was 0.806, indicating acceptable internal consistency.

### 2.4 Quality control

Clinical assessment data were extracted from the electronic medical record system and nursing records, including questionnaire scores and serum test results obtained before radiotherapy and 30 days after its completion. All data were independently extracted and entered by two investigators and cross-checked to ensure accuracy and completeness. Missing or abnormal data were further reviewed and addressed to ensure the reliability of the analysis.

### 2.5 Statistical analysis

All statistical analyses were performed using R software (version 4.5.1). Because all outcomes were assessed in the same individuals before and after radiotherapy, paired statistical tests were used throughout. A two-sided p value <0.05 was considered statistically significant. Continuous variables were presented as mean ± standard deviation. Paired t-tests were employed for comparisons of normally distributed continuous variables before and after radiotherapy, whereas the Wilcoxon signed-rank test was used for non-normally distributed continuous variables and ordinal data. Pearson correlation analysis was performed to investigate the relationships among sexual function scores, sex hormone levels, and psychological variables.

## 3 Results

### 3.1 Baseline characteristics of the study population

A total of 90 male patients with nasopharyngeal carcinoma who met the inclusion criteria were screened, of whom 85 with complete data were included in the final analysis. All patients were married and lived with their spouses, and none reported religious beliefs. The mean age was 45.80 ± 9.84 years. Regarding educational level, 11 patients had completed primary school, 42 junior high school, 19 senior high school or technical secondary school, and 13 had college education or above. According to disease stage, 3 patients were classified as stage I, 9 as stage II, 27 as stage III, and 45 as stage IVa.

### 3.2 Sexual function changes prior to and following radiation therapy

Both erectile and ejaculatory function scores showed significant reductions after radiotherapy. Compared with baseline, the International Index of Erectile Function-5 (IIEF-5) score and the CIPE score were significantly decreased after treatment (both P < 0.001). The effect sizes were r = 0.757 for the IIEF-5 score and r = 0.737 for the CIPE score, indicating a marked decline in sexual function. Further correlation analysis showed a strong positive correlation between IIEF-5 and CIPE scores (r = 0.778).

### 3.3 Changes in HPG axis–related hormone levels before and after radiotherapy

Comparisons of hypothalamic–pituitary–gonadal axis–related hormone levels showed significant changes after radiotherapy. Follicle-stimulating hormone (FSH) and prolactin (PRL) levels were significantly increased compared with baseline, whereas testosterone (T) and progesterone (P) levels were significantly decreased after treatment (all P < 0.001). The corresponding effect sizes were r = 0.567 for FSH, r = 0.462 for PRL, d = 0.446 for T, and r = 0.378 for P. In contrast, no statistically significant differences were observed in luteinizing hormone (LH) or estradiol (E2) levels between pre- and post-radiotherapy measurements (both P > 0.05).

### 3.4 Psychological status changes prior to and following radiation therapy

Comparisons of psychological status before and after radiotherapy showed no statistically significant differences in anxiety or depression levels. Scores on the Self-Rating Anxiety Scale (SAS) and the Self-Rating Depression Scale (SDS) did not change significantly between the two time points (both P > 0.05), indicating that overall psychological status remained relatively stable during the acute phase after radiotherapy, with most patients presenting mild levels of anxiety and depression.

### 3.5 Relationships between sexual hormones, psychological status, and sexual function

Correlation analyses showed weak associations between post-radiotherapy sexual function scores (IIEF-5 and CIPE) and hypothalamic–pituitary–gonadal axis–related hormone parameters (|r| < 0.3). A strong positive correlation was observed between IIEF-5 and CIPE scores (r = 0.778), while SAS and SDS scores showed a moderate positive correlation (r = 0.348).

Among sex hormone parameters, prolactin (PRL) was strongly negatively correlated with testosterone (T) levels (r = −0.868). In addition, follicle-stimulating hormone (FSH), luteinizing hormone (LH), testosterone (T), and estradiol (E2) were generally positively correlated with each other, whereas PRL was negatively correlated with most of these hormone variables.

## 4 Discussion

In this retrospective analysis of 85 male patients with nasopharyngeal carcinoma who underwent intensity-modulated radiotherapy (IMRT), a significant decline in sexual function scores was observed within 30 days after treatment. A strong positive correlation between erectile function (IIEF-5) and ejaculatory function (CIPE) scores suggested that sexual dysfunction occurred early in the acute phase and showed consistent patterns across functional domains. In contrast, no significant changes were observed in anxiety (SAS) or depression (SDS) scores before and after radiotherapy. Correlation analyses further showed weak associations between post-radiotherapy sexual function and hypothalamic–pituitary–gonadal (HPG) axis–related hormone levels, and no consistent linear relationships were identified between psychological status and sexual function. These findings suggest that sexual dysfunction in male patients with nasopharyngeal carcinoma during the acute phase of IMRT is more likely to be related to organic and neuroendocrine dysregulation, while psychological factors may play a relatively limited role at this stage. In this study, sexual function primarily refers to erectile and ejaculatory function, as other domains such as libido, orgasm, and sexual satisfaction were not assessed.

### 4.1 Clinical features of sexual dysfunction in male patients with nasopharyngeal cancer during the acute phase of IMRT

Our data show that male patients with nasopharyngeal carcinoma exhibit a significant decline in sexual function during the acute phase following IMRT. Previous studies have consistently reported reduced sexual function in patients with head and neck cancers after radiotherapy. A prospective study of patients with head and neck cancer demonstrated a significant decrease in sexual function scores within three months after radiotherapy, confirming the acute adverse effects of radiotherapy on sexual function (16). However, that study included multiple tumor sites and treatment regimens, which may have introduced heterogeneity into the findings.

In contrast, the present study focused on male patients with nasopharyngeal carcinoma, with relatively homogeneous clinical characteristics in terms of age, disease stage, and treatment modality. This more homogeneous population allows for a clearer assessment of the acute effects of IMRT on sexual function. Consistent with previous findings, we observed a significant decline in sexual function scores within 30 days after radiotherapy. These findings suggest that even under modern IMRT techniques, sexual dysfunction remains a clinically relevant issue during the acute phase of nasopharyngeal carcinoma and should not be overlooked.

### 4.2 Acute effects of IMRT on hypothalamic–pituitary–gonadal axis–related hormones

Male patients with nasopharyngeal carcinoma showed asynchronous changes in hypothalamic–pituitary–gonadal (HPG) axis–related hormone levels after intensity-modulated radiotherapy. Specifically, post-radiotherapy assessments indicated a dysregulated hormonal pattern characterized by significant increases in follicle-stimulating hormone (FSH) and prolactin (PRL), accompanied by significant decreases in testosterone (T) and progesterone (P). In contrast, luteinizing hormone (LH) and estradiol (E2) showed increasing and decreasing trends, respectively, without reaching statistical significance.

In the present study, FSH levels increased significantly after radiotherapy, whereas LH levels showed only a slight and non-significant increase. Previous studies have suggested that the frequency of gonadotropin-releasing hormone (GnRH) pulses plays a key role in regulating pituitary gonadotropin secretion, with high-frequency GnRH pulses preferentially promoting LH release and low-frequency pulses favoring FSH secretion (17). Radiation-induced injury to the hypothalamus and pituitary may reduce GnRH pulse frequency, thereby promoting FSH secretion while relatively suppressing LH release.

Furthermore, despite the absence of a corresponding decrease in LH, PRL levels increased significantly, accompanied by a marked reduction in T levels. This pattern suggests that post-radiotherapy hyperprolactinemia may result from radiation-induced disruption of hypothalamic dopaminergic pathways, leading to disinhibition of PRL secretion (18). Previous studies have shown that elevated PRL levels can directly impair Leydig cell function via activation of the JAK2–STAT5 signaling pathway, thereby reducing progesterone and testosterone production (19). Notably, the observed decrease in progesterone represents a novel finding in this study. Although mean progesterone levels remained within the clinically normal range, its potential role as an early indicator of impaired steroidogenesis requires further investigation. Given that testosterone serves as a precursor for estradiol synthesis, the reduction in T levels may partly explain the downward trend observed in E2 levels.

### 4.3 Possible mechanisms behind the limited correlation between sexual dysfunction and HPG axis hormone levels

In the present study, sexual function scores during the acute phase after radiotherapy showed weak associations with multiple hypothalamic–pituitary–gonadal (HPG) axis hormone levels. Whether radiotherapy-related sexual dysfunction is primarily mediated by alterations in gonadal hormone levels remains inconclusive. Previous studies have also reported no significant associations between sexual function scores and serum levels of testosterone, luteinizing hormone, follicle-stimulating hormone, or prolactin (20), suggesting that the traditional “sex hormone–driven” mechanism may not be the sole or predominant pathway underlying radiotherapy-associated sexual dysfunction.

Consistent with these findings, our results further support the notion that changes in sexual function cannot be fully explained by alterations in individual hormone levels alone. Instead, sexual dysfunction following radiotherapy may involve more complex neuroendocrine regulatory mechanisms (21), rather than simple linear hormone–response relationships. In addition, the relatively young age of the study population (mean age, 45.8 years) and the short interval between radiotherapy completion and evaluation may indicate that compensatory endocrine regulation remains active during the acute phase. These adaptive mechanisms may partially attenuate the direct impact of hormone level changes on sexual function.

### 4.4 Role of psychological variables in sexual dysfunction during the acute phase of IMRT

During the acute phase of intensity-modulated radiotherapy, no significant changes were observed in anxiety (SAS) or depression (SDS) scores before and after treatment, and overall psychological status was mainly characterized by mild anxiety and depression. These findings suggest that psychological status remains relatively stable during this stage, and that the occurrence of sexual dysfunction in male patients with nasopharyngeal carcinoma is not necessarily accompanied by a marked deterioration in emotional status.

Notably, correlation analysis after radiotherapy showed a negative association between depression scores (SDS) and sexual function, indicating that, at the individual level, depressive symptoms may still influence male sexual function to some extent. Previous studies have reported that severe depressive states are significantly associated with male sexual dysfunction through effects on sexual desire, erectile function, and sexual satisfaction (22). However, those studies mainly involved patients with major depressive disorder, whose symptom severity and duration were substantially greater than those observed in the present cohort, which was predominantly characterized by mild depressive symptoms. This difference suggests that such findings may not be directly generalizable across different disease stages and psychological conditions.

Several factors may account for the limited influence of psychological variables observed in this study. First, all included patients had Karnofsky Performance Status scores >90 and no severe physical or psychiatric comorbidities, indicating relatively preserved functional status and psychological resilience, which may attenuate the impact of acute emotional fluctuations on sexual function. Second, within the Chinese cultural context, male patients may exhibit social desirability bias when reporting sensitive issues related to sexual function and psychological well-being, potentially leading to underreporting of emotional distress (23).This phenomenon was also supported by post-study interviews with a subset of participants.

This study has several limitations. First, this was a single-center retrospective study with a relatively small sample size, which may introduce selection bias. Second, the follow-up period was limited to 30 days after radiotherapy, and longer-term outcomes were not assessed. Third, most patients received concurrent chemoradiotherapy, and no stratified analysis according to treatment modality was performed, which may have influenced the results. Fourth, potential differences in target delineation and dose distribution across disease stages were not further explored through subgroup analyses. In addition, dosimetric evaluation of the hypothalamic–pituitary region was not performed, and its role in endocrine alterations requires further investigation. Finally, the inclusion of only married patients living with their spouses, as well as those whose acute treatment-related adverse effects had largely resolved at the time of assessment, may have improved the consistency of evaluation but could limit the generalizability of the findings.

## 5 Conclusion

Intensity-modulated radiotherapy was associated with a significant decline in sexual function among male patients with nasopharyngeal carcinoma during the acute phase, accompanied by alterations in hypothalamic–pituitary–gonadal axis–related hormone levels. In clinical practice, risk communication may be strengthened before treatment, and sexual function assessment may be incorporated into follow-up care. For patients with relevant symptoms, combined endocrine evaluation and targeted health education may be considered.

## Data Availability

The raw data supporting the conclusions of this article will be made available by the authors, without undue reservation.
