# Supplementary material for: Acute changes in sexual function, hormonal profiles, and psychological status following intensity-modulated radiotherapy in male patients with nasopharyngeal carcinoma
Source: Front Oncol. 2026 Jun 2;16:1832794. doi: 10.3389/fonc.2026.1832794 (PMC13268945; doi:10.3389/fonc.2026.1832794)

Supplementary Material

Questionnaires Used in This Study

**Supplementary Material 1.** International Index of Erectile Function-5 (IIEF-5).The IIEF-5 questionnaire is reproduced from Rosen et al. (1999).


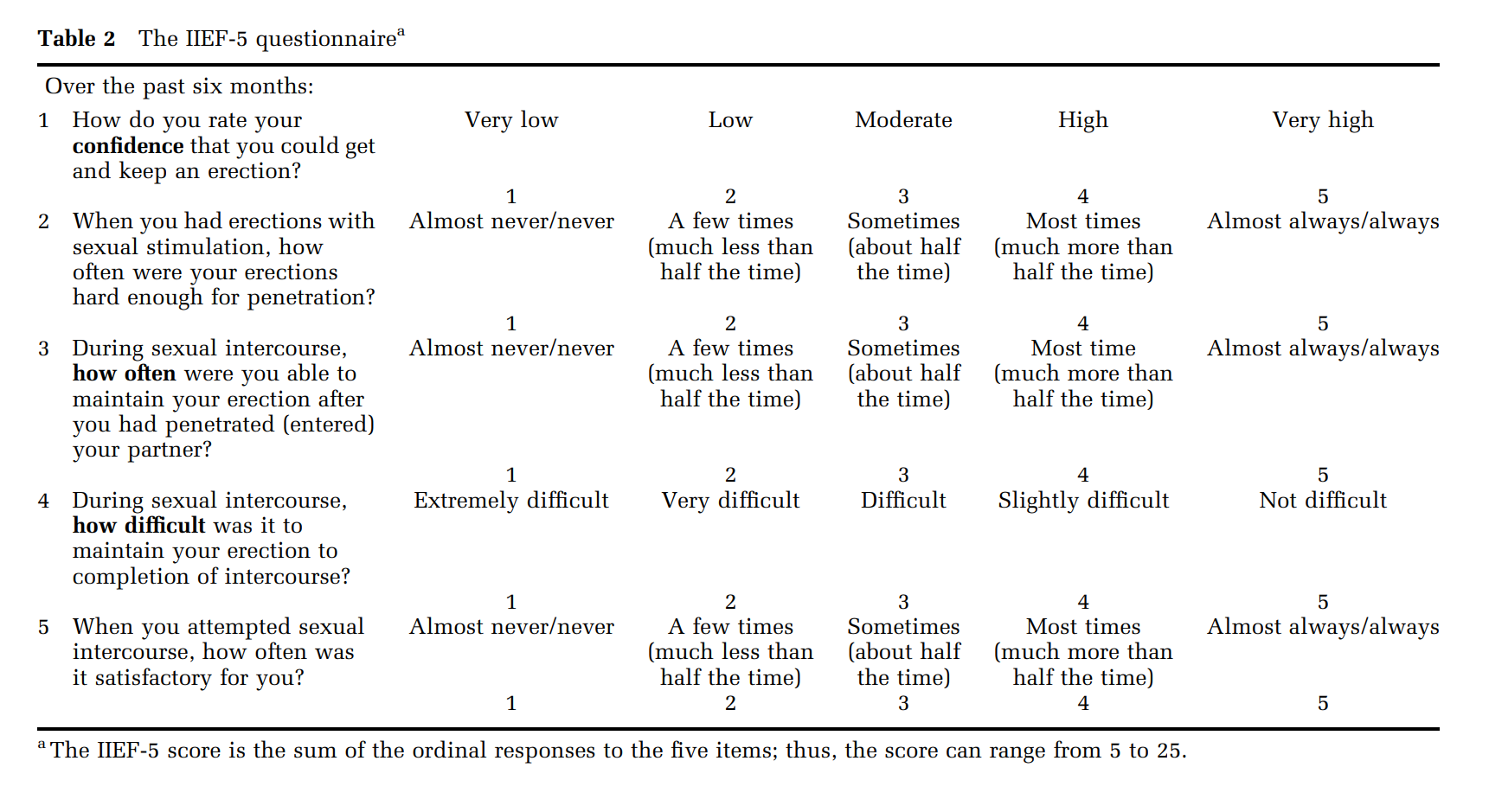


**Supplementary Material 2.** Chinese Index of Sexual Function for Premature Ejaculation (CIPE)
The CIPE questionnaire was adapted from Yuan et al. (2003) and used to assess ejaculatory function and related aspects of sexual function in male patients.
Instructions:
Please answer the following questions based on your sexual experience over the past 6 months.

Q1. How would you rate your level of sexual desire?

1 = Very low

2 = Low

3 = Moderate

4 = High

5 = Very high

Q2. During sexual activity, how often was your erection sufficient for vaginal penetration?

1 = Almost never/never

2 = A few times (much less than half the time)

3 = Sometimes (about half the time)

4 = Most times (much more than half the time)

5 = Almost always/always

Q3. During sexual intercourse, how often were you able to maintain your erection until completion?

1 = Almost never/never

2 = A few times (much less than half the time)

3 = Sometimes (about half the time)

4 = Most times (much more than half the time)

5 = Almost always/always

Q4. What was your typical ejaculatory latency time (from penetration to ejaculation)?

1 = Very short (<30 seconds)

2 = Short (~1 minute)

3 = Moderate (~2 minutes)

4 = Longer (~3 minutes)

5 = Long (>5 minutes)

Q5. How difficult was it for you to control ejaculation during intercourse?

1 = Extremely difficult

2 = Very difficult

3 = Difficult

4 = Slightly difficult

5 = Not difficult

Q6. Overall, how satisfied are you with your sexual life?

1 = Very dissatisfied

2 = Dissatisfied

3 = Neutral

4 = Satisfied

5 = Very satisfied

Q7. Overall, how satisfied is your partner with your sexual life?

1 = Very dissatisfied

2 = Dissatisfied

3 = Neutral

4 = Satisfied

5 = Very satisfied

Q8. How often does your partner achieve orgasm during sexual activity?

1 = Almost never/never

2 = A few times

3 = Sometimes

4 = Most times

5 = Almost always/always

Q9. How confident are you in successfully completing sexual intercourse?

1 = Very low

2 = Low

3 = Moderate

4 = High

5 = Very high

Q10. During sexual activity, how often do you feel anxious or tense?

1 = Almost always

2 = Most times

3 = Sometimes

4 = A few times

5 = Almost never

Scoring:
Each item is scored from 1 to 5. Total score ranges from 10 to 50. Lower scores indicate worse ejaculatory function.
Reference:
Yuan Y, Xin Z, Jin T, et al. Chinese index of sexual function for premature ejaculation (CIPE). Chinese Journal of Andrology. 2003.
Note:
All questionnaires were used according to their original published versions. Appropriate references are provided.

**Supplementary Material 3.** Self-Rating Anxiety Scale (SAS)

The SAS questionnaire is reproduced from Zung (1971).


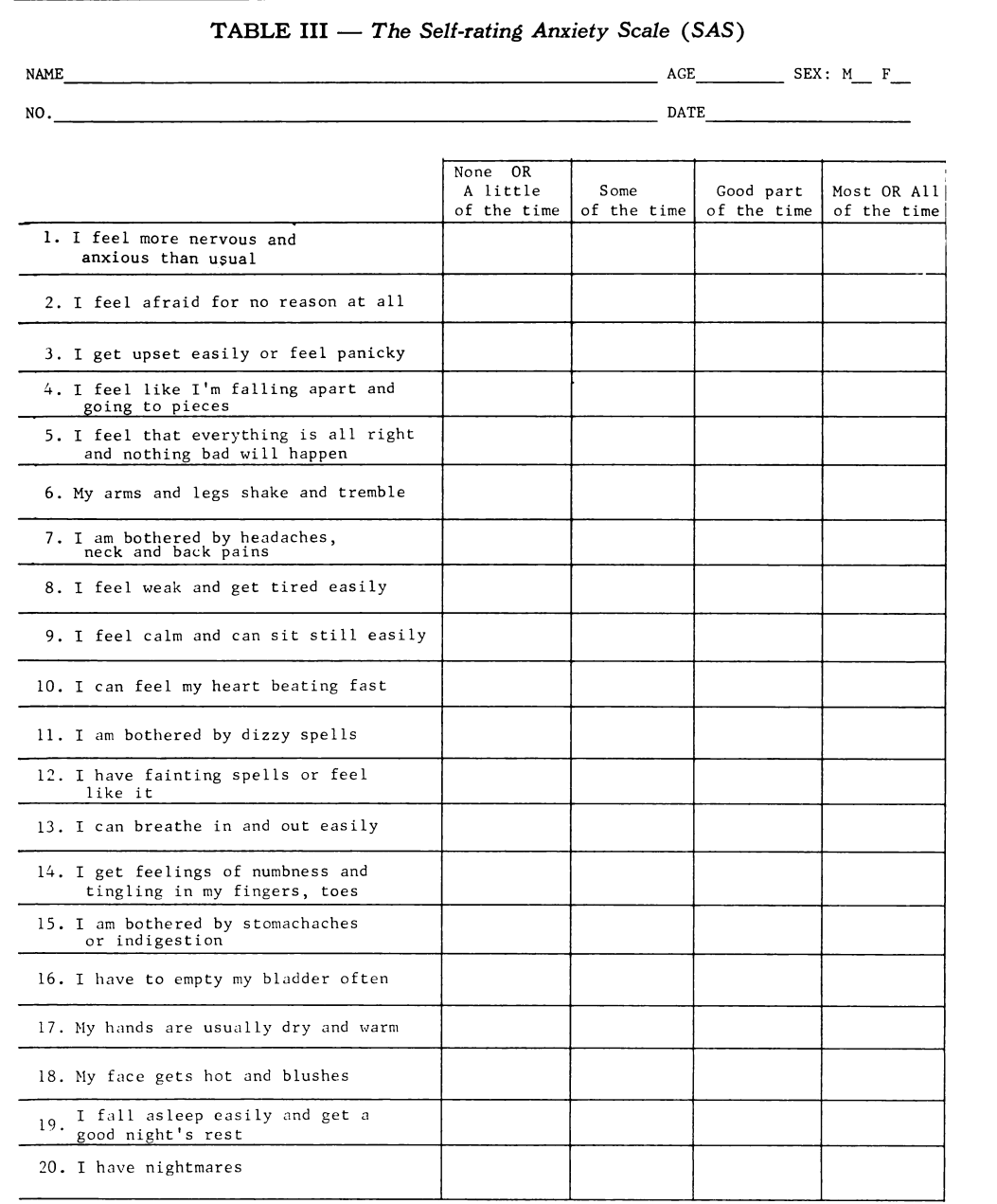


**Supplementary Material 4.** Self-Rating Depression Scale (SDS)

The SDS questionnaire is reproduced from Zung (1965).


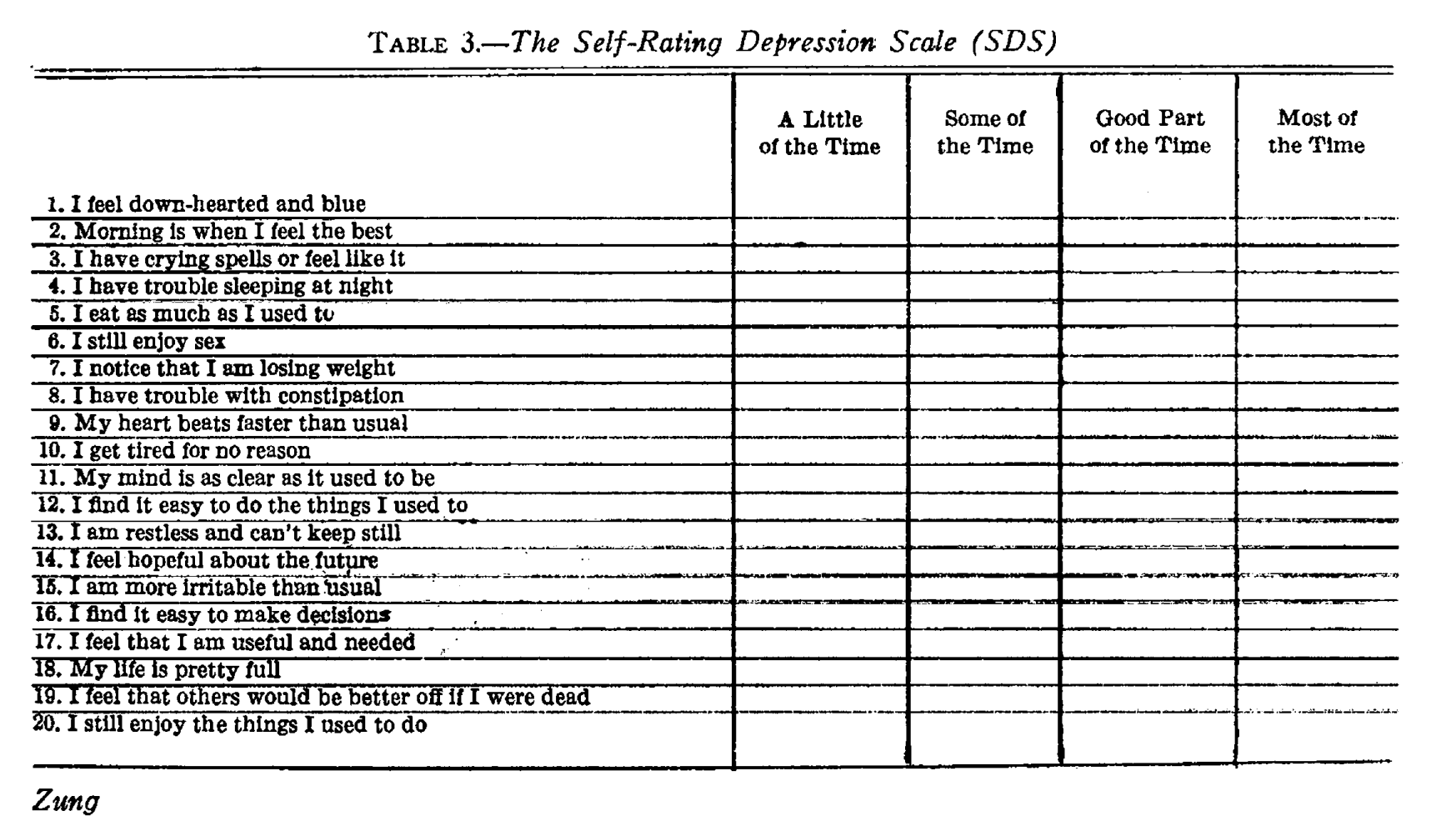

Supplement: Supplementary file 1 [file DataSheet1.docx]
